# Three-Dimensional Modeling of Thyroid Hormone Metabolites Binding to the Cancer-Relevant αvβ3 Integrin: *In-Silico* Based Study

**DOI:** 10.3389/fendo.2022.895240

**Published:** 2022-05-27

**Authors:** Dror Tobi, Eilon Krashin, Paul J. Davis, Vivian Cody, Martin Ellis, Osnat Ashur-Fabian

**Affiliations:** ^1^Department of Molecular Biology, Ariel University, Ariel, Israel; ^2^Department of Computer Sciences, Ariel University, Ariel, Israel; ^3^Translational Oncology Laboratory, Meir Medical Center, Kfar-Saba, Israel; ^4^Department of Human Molecular Genetics and Biochemistry, Sackler School of Medicine, Tel Aviv University, Tel Aviv, Israel; ^5^Department of Medicine, Albany Medical College, Albany, NY, United States; ^6^Pharmaceutical Research Institute, Albany College of Pharmacy and Health Sciences, Albany, NY, United States; ^7^Hauptman-Woodward Medical Research Institute & Department of Structural Biology, SUNY, University at Buffalo, Buffalo, NY, United States; ^8^Hematology Institute and Blood Bank, Meir Medical Center, Kfar-Saba, Israel; ^9^Sackler School of Medicine, Tel Aviv University, Tel Aviv, Israel

**Keywords:** thyroid hormones, binding energy, integrin, affinity, *in-silico* docking

## Abstract

**Background:**

Thyroid hormones (TH), T4 and T3, mediate pro-mitogenic effects in cancer cells through binding the membrane receptor αvβ3 integrin. The deaminated analogue tetrac effectively blocks TH binding to this receptor and prevents their action. While computational data on TH binding to the αvβ3 integrin was published, a comprehensive analysis of additional TH metabolites is lacking.

**Methods:**

*In-silico* docking of 26 TH metabolites, including the biologically active thyroid hormones (T3 and T4) and an array of sulfated, deiodinated, deaminated or decarboxylated metabolites, to the αvβ3 receptor binding pocket was performed using DOCK6, based on the three-dimensional representation of the crystallographic structure of the integrin. As the TH binding site upon the integrin is at close proximity to the well-defined RGD binding site, linear and cyclic RGD were included as a reference. Binding energy was calculated for each receptor-ligand complex using Grid score and Amber score with distance movable region protocol.

**Results:**

All TH molecules demonstrated negative free energy, suggesting affinity to the αvβ3 integrin. Notably, based on both Grid and Amber scores sulfated forms of 3,3’ T2 (3,3’ T2S) and T4 (T4S) demonstrated the highest binding affinity to the integrin, compared to both cyclic RGD and an array of examined TH metabolites. The major thyroid hormones, T3 and T4, showed high affinity to the integrin, which was superior to that of linear RGD. For all hormone metabolites, decarboxylation led to decreased affinity. This corresponds with the observation that the carboxylic group mediates binding to the integrin pocket *via* divalent cations at the metal-ion-dependent adhesion (MIDAS) motif site. A similar reduced affinity was documented for deaminated forms of T3 (triac) and T4 (tetrac). Lastly, the reverse forms of T3, T3S, and T3AM showed higher Amber scores relative to their native form, indicating that iodination at position 5 is associated with increased binding affinity compared to position 5’.

**Summary:**

Three-dimensional docking of various TH metabolites uncovered a structural basis for a differential computational free energy to the αvβ3 integrin. These findings may suggest that naturally occurring endogenous TH metabolites may impact integrin-mediate intracellular pathways in physiology and cancer.

## Introduction

Thyroid hormones (THs) are essential for the normal development of tissues, as well as for the regulation of cellular metabolism, cell structure and membrane transport (1). Biosynthesis of THs requires several coordinated steps. The prohormone 3,5,3’,5’ -tetraiodothyronine (T4) is synthesized on thyroglobulin (Tg) in thyroid follicles (2). Iodination of tyrosyl residues and phenolic coupling of the iodotyrosyl residues on Tg by thyroid peroxidase (TPO) forms T4, followed by its proteolytic liberation (3). After delivery to target tissues, T4 undergo deiodination by iodothyronine deiodinases (DIO1, DIO2, and DIO3) to a variety of active and inactive metabolites (4). The removal of one iodine atom from the phenolic (outer) ring of T4 produces the biologically active hormone 3,5,3’-triodothyronine (T3), whereas removal of an iodine atom from the tyrosyl (inner) ring leads to the formation of a biologically inactive metabolite, 3,3’,5’-triodothyronine also known as reverse T3 (rT3). DIO1 converts T3 into 3,5 diiodothyronine (3,5 T2), while DIO1/DIO3 converts T3 into 3,3’ T2. DIO1 and DIO2 also produce 3,3’- T2 by deiodination of the phenolic ring of rT3. Both 3,5 T2 and 3,3’ T2 display thyromimetic activity (5, 6). A series of further deiodinations produces 3’-T1 and T0. THs undergo additional metabolic modifications. The conjugation of the phenolic hydroxy group (4’-OH) of T3 and T4 with sulfate and glucuronic acid yields the corresponding sulfated and glucoronidated hormones (7), serving as inactivating pathways of TH action and enhancing their excretion (8, 9). T4 and T3 may further undergo oxidative deamination by transaminase and l-amino acid oxidase to produce their corresponding iodothyroacetic acids, tetrac and triac. Another potential modification, is the removal of the carboxyl group of thyroid hormones (decarboxylation) producing iodothyronamines (TAMs). TAMs may further undergo oxidative deamination by monoamine oxidase to produce the iodothyroacetic acids metabolites. Ether link cleavage by horseradish peroxidase, myeloperoxidase and TPO catalyzes the conversion of T4 into 3,5-diiodotyrosine (DIT) (10–12). The iodine atom from TAMs, iodothyroacetic acids and sulfated THs can be recycled through deiodination (13). TH deiodination and the various metabolic alterations were reviewed and illustrated (14).

TH effects depend on the transcriptional modulation of specific genes, which is mediated *via* nuclear uptake of the biologically active hormone T3, the formation of complexes between T3 and nuclear thyroid hormone receptor (TR) proteins, and the subsequent occupancy of regulatory complexes at thyroid hormone response elements on hormone-responsive genes (15–17). However, data presented in recent decades support the existence of a number of nongenomic mechanisms of action for the hormone (18, 19). In 2005, a cell surface receptor for thyroid hormone has been identified on the extracellular domain of the *α*v*β*3 integrin (20). Integrins are a family of 24 structural proteins found in plasma membranes and are essential regulators of cell–cell and cell–extracellular matrix (ECM) protein interactions (21). Integrin αvβ3 is one of eight integrins with an Arg–Gly–Asp (RGD) recognition site that binds ECM proteins containing this sequence, such as vitronectin, fibronectin and osteopontin (21). The X-ray structure of *α*v*β*3 has been determined in the presence of Mn^2+^ as a complex with a cyclic RGD peptide (22). Structural data have revealed that the extracellular segment of integrin *α*v*β*3 is V shaped with the 4-domain *α*v subunit and the 8-domain *β*3 subunit bent by 135*◦* (22–24). The RGD peptide occupies a shallow crevice between the propeller and *β*A domains in the integrin head. A common structural motif observed for integrins is the presence of a metal binding site, termed metal ion-dependent adhesion site (MIDAS) in the integrin *α* subunit I domain, which binds the divalent cations Mg^2+^/Mn^2+^ and Ca^2+^. This site is essential not only for the formation of the integrin heterodimer but also for bridging ligand binding to the integrin. Generally, ligand binding is stimulated by Mg^2+^ or Mn^2+^ and inhibited by Ca^2+^ (25–27).

Subjected to crystallographic modeling (28) and mathematical modelling of the kinetics of thyroid hormone-binding (29) to the extracellular fragment of *α*v*β*3 integrin, the thyroid hormone receptor on integrin αvβ3 has been shown to consist of two binding domains. The S1 domain exclusively recognizes T3 and activates PI3K *via* Src kinase. The S2 domain regulates MAPK1 and MAPK2 and binds both T4 and T3. The S2 domain has a higher affinity for T4 than the S1 or S2 sites have for T3. A trophic effect of T4 mediated through the membrane integrin αvβ3 has been shown experimentally *in vitro* and *in vivo* in cancer cells, leading to induction of proliferation (19, 20, 30–34) and inhibition of apoptosis (32). Tetrac, the deaminated analogue of T4, blocks thyroid hormone-binding at the integrin and inhibits the ability of thyroid hormone analogues to activate MAPK (20). Competition data revealed that RGD peptides also block hormone binding by integrin, suggesting that the hormone-binding site is near the RGD recognition site on *α*v*β*3 (27, 35).

In recent decades, advances in computational methodologies have enabled virtual docking of small molecules to target sites. Using these methods, ligand conformation that best matches the receptor structure is identified, followed by evaluation of energy of binding (36). The calculated free energy serves as a quantitative predictor of ligand binding affinity (37). In this work, using *in-silico* docking to the *α*v*β*3 integrin receptor, binding affinity was assessed for 26 thyroid hormones and their metabolites.

## Methods

A library of 26 thyroid hormones and their metabolites were defined (**Supplementary Figure S1**). These include all active forms of thyroid hormones as well as deiodinated, sulfated, decarboxylated and deaminated metabolites. Three dimensional representations of ligands at physiologic pH were retrieved from the ZINC15 compound database (38), except for reverse T3 sulfate which was retrieved from Pubchem (https://pubchem.ncbi.nlm.nih.gov/compound/133192#section=3D-Conformer). Linear RGD and cyclic RGD (cRGD) were used as a reference. *In-silico* docking of molecules to the αvβ3 receptor RGD binding pocket was performed, based on the three dimensional crystal structure of the integrin (PDB code1L5G) (23). Briefly, orientations of the ligand relative to a receptor binding cavity were searched using a negative surface image of the target. The image was generated by filling the solvent accessible receptor surface with overlapping spheres and selecting a subset of the spheres to represent the binding site (39). Ligand atoms and spheres were geometrically matched to sample rigid-body orientational space (40), and conformational space of the ligand were sampled in the presence of the receptor binding site *via* the anchor-and-grow incremental construction approach (41) and a grid based score (Grid score) is calculated for each docked molecule (kcal/mol). Next, a more elaborate free energy rescoring step was carried out for each receptor-ligand complex using Amber score. This technique uses the generalized Born/surface area (GB/SA) continuum model for solvation free energy (kcal/mol). Therefore, it provides good estimation of the free energy of the molecules to the receptor with the limitation that configurational entropy effects are ignored. The Amber score is calculated as EComplex − (EReceptor + ELigand), where EComplex, EReceptor, and ELigand are molecular mechanics energies combined with the generalized Born and surface area continuum solvation energies (MM-GB/SA) as approximated by the Amber force field (42). The ligand and residues within 4Å from the ligand were defined as fully flexible within the ligand-receptor complex, allowing small structural rearrangements to reproduce the so-called “induced fit” (43) while performing the scoring. Docking and scoring was performed using UCSF DOCK6 software (44). Amber score with distance movable protocol and the following parameters were used: amber_score_before_md_minimization_cycles 200, amber_score_md_steps 6000 amber_score_after_md_minimization_cycles 200 doubling the parameters recommended by Brozell et al. to ensure sufficient calculation time (45). Magnesium (Mg^2+^) atoms were used for MIDAS sites, as its force field parameters are included in DOCK6 package. Protonation state of histidine and other titratable residues was determined using PDB2PQR and if needed amino acids contacting the Mg^2^+ were set to their acidic state manually (46). Docked structure was prepared and results were inspected using UCSF Chimera, a visualization system for exploratory research and analysis (47).

## Results

**Figure 1:**
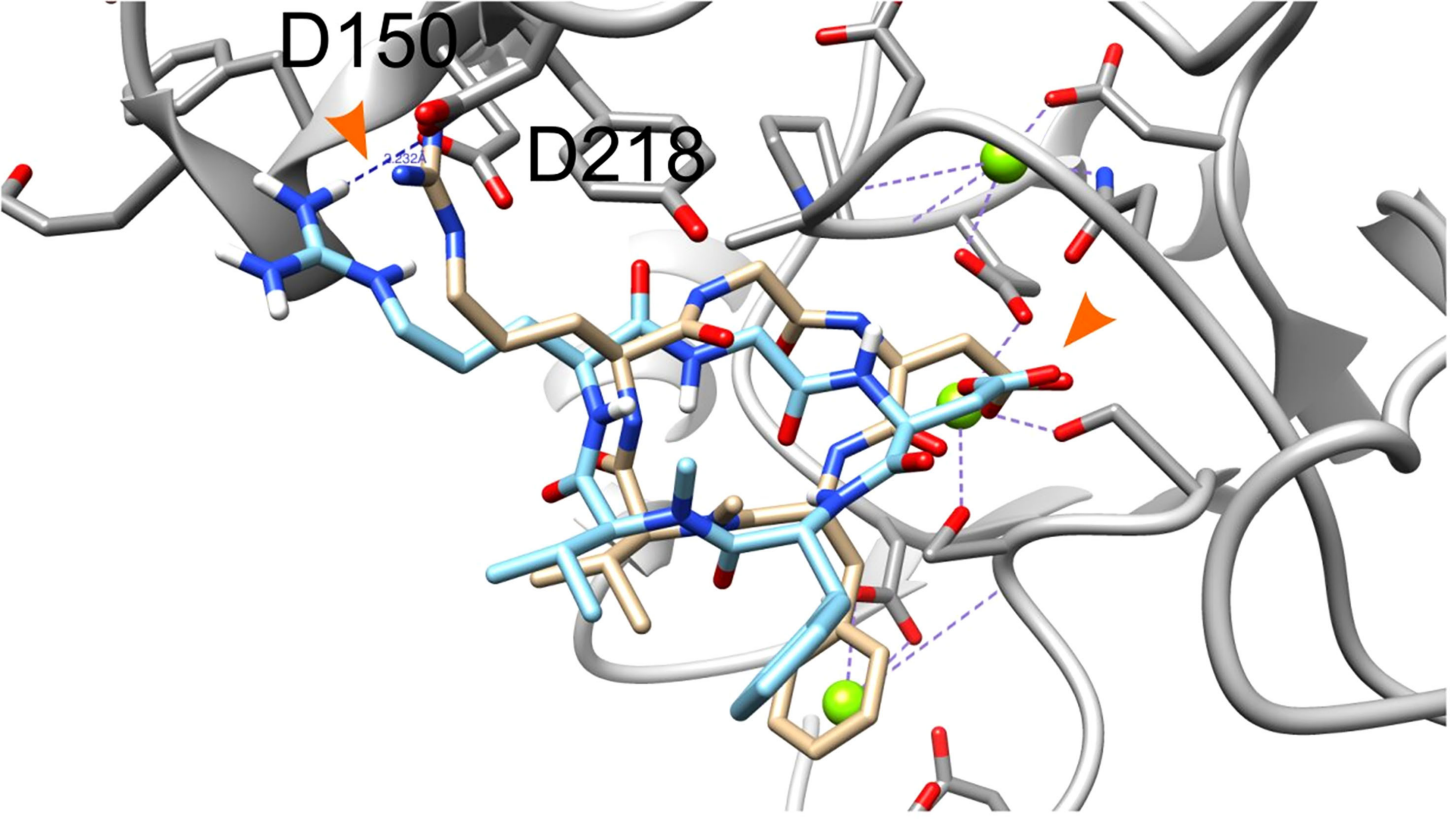
Docking of the cyclic pentapeptide ligand Arg-Gly-Asp-[D-Phe]-[N-methyl-Val-] to integrin αVβ3. Integrin’s binding site backbone (ribbons) and side chains (sticks) are colored gray with oxygen atoms in red and nitrogen in blue. The crystallography and docked structures of the cyclic peptide Arg-Gly-Asp-[D-Phe]-[*N-methyl*-Val-] are shown using stick representations with carbon atoms colored gold and cyan, respectively. Oxygen, nitrogen, and hydrogen atoms are colored red, blue and white, respectively. Mg^2+^ atoms are shown as green spheres. Ligand side chains Arg and Asp are marked with orange arrowhead.

The crystal structure of extracellular segment of the integrin αvβ3, in complex with cyclic pentapeptide ligand Arg-Gly-Asp-[D-Phe]-[*N-methyl*-Val-] that mimics the natural Arg-Gly-Asp ligand, was used for the docking calculations. Evaluation of the docking procedure was carried out by docking the cyclic pentapeptide ligand to its binding site in the integrin. **Figure 1** presents the crystallographic structure of the Arg-Gly-Asp ligand (carbon atoms colored gold) in the integrin αvβ3 binding site vs. the docked structure (carbon atoms colored cyan). The Arg and Asp ligand side chains are marked with orange arrowhead. The carboxyl groups of crystallographic and docked structure are almost overlapping and interact with the Mg^2+^ atoms (green sphere). Both arginine side chains of the crystallographic and docked structure contact Asp218 and their nitrogen atoms are 3.6Å apart. The calculated docking Grid score is -81.85 and the rescoring step with Amber force field resulted in Amber score of -58.30. The negative scores indicate good affinity of the RGD ligand to the integrin binding site. In comparison the cyclic RGD, the linear RGD molecule displays a significantly lower amber score (-33.82). The overall good overlap between the crystallographic and docked structure and the accepted scores indicate the ability of the DOCK6 package and the parameters we used to correctly dock and score ligand to the integrin binding site.

**Figure 2:**
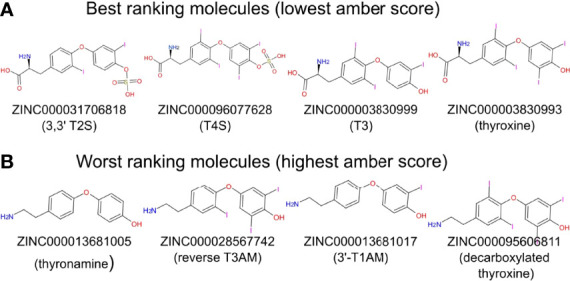
Two-dimensional structure of molecules having the best and worst Amber score. **(A)** The four molecules showing the best (lowest) Amber score. **(B)** The four molecules showing the worst (highest) Amber score.

**Figure 3:**
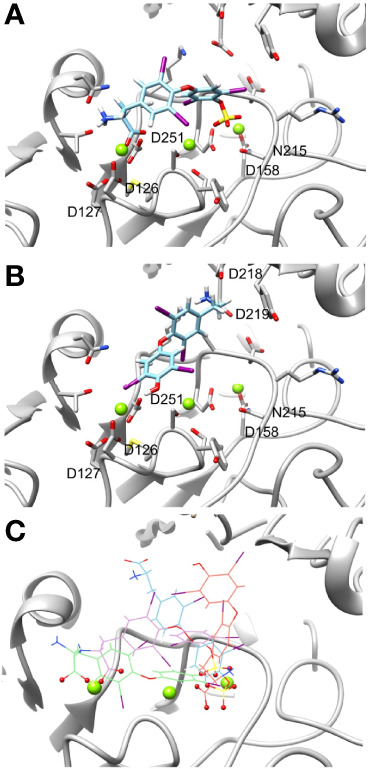
Docking structures of high and low scoring molecules in the integrin αvβ3 RGD binding site. Integrin’s binding site backbone (ribbons) and side chains (sticks) are colored gray with oxygen atoms in red and nitrogen in blue. **(A)** Stick representation of T4S (ZINC000096077628) docked pose in the RGD binding site. T4S form electrostatic interaction with one Mg^2+^ atom of the MIDAS sites through the carboxylic group and with another Mg^2+^ atom through the sulfate group. **(B)** T4AM (decarboxylated thyroxine, ZINC000095606811) docked pose does not form electrostatic interactions with Mg^2+^ atoms. **(C)** Docked poses of the four molecules showing the lowest Amber score. The molecules are depicted using wire representation and the negatively charged oxygens of the carboxylic and sulfate groups are presented as red spheres. The Mg^2+^ atom is shown as green sphere and docked structures are colored as follows: 3,3’ T2S carbon (cyan), T4S carbon (orchid), T3 carbon (green), thyroxine (orange), nitrogen (blue), oxygen (red), hydrogen (white), iodine (purple), and sulfur (yellow). For clarity the oxygen atoms are presents as smaller spheres compared to the Mg^2+^ atoms although their actual atomic radius is larger.

**Figure 4:**
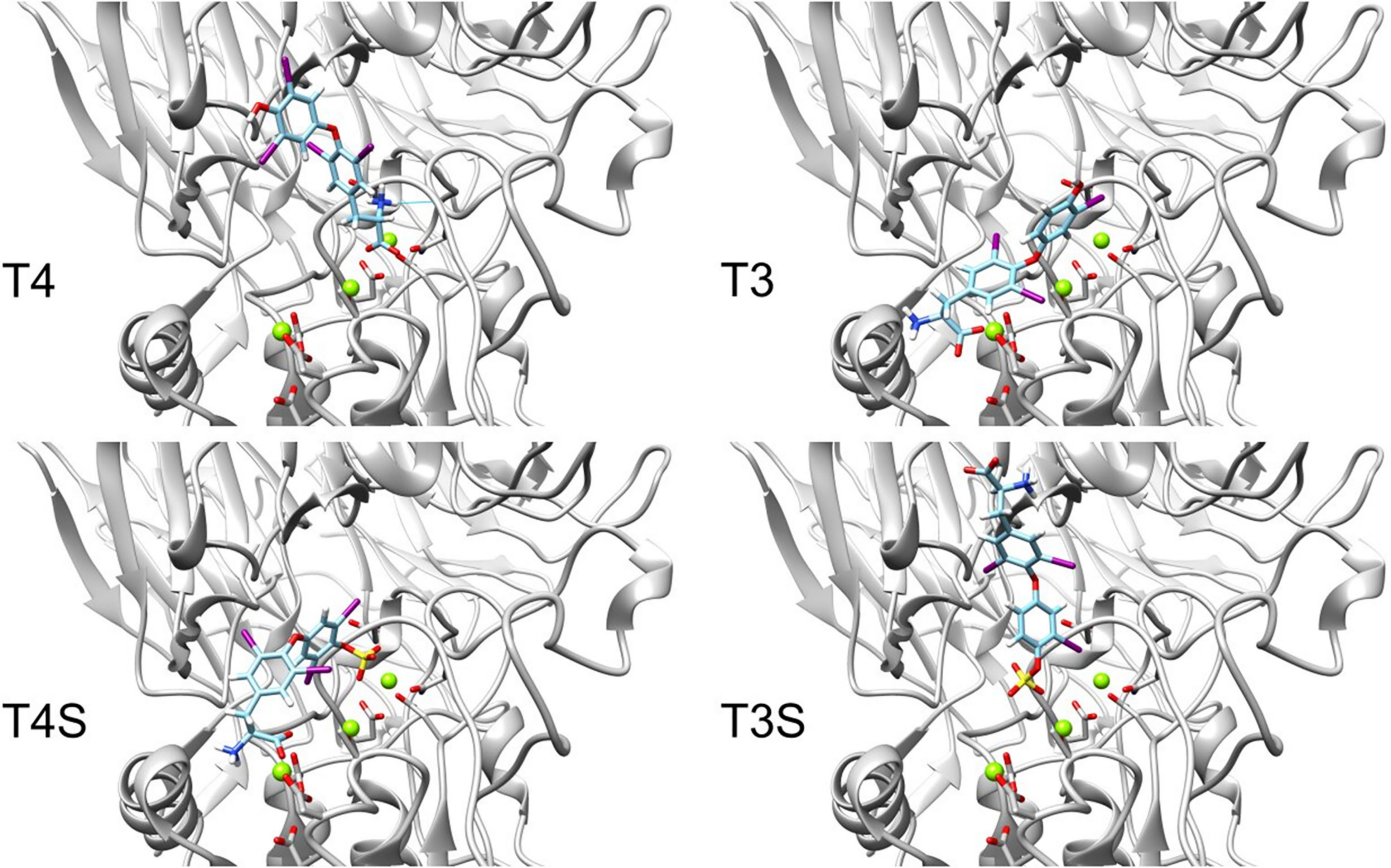
Docking structures of T4, T3 to the integrin αvβ3 binding site in comparison to their sulfated forms. Integrin’s binding site backbone (ribbons) and side chains (sticks) are colored gray with oxygen atoms in red and nitrogen in blue. Comparison between the docked structure of T3 (ZINC000003830999), T4 (ZINC000003830993), sulfated T3 (ZINC000031706818) and sulfated T4 (ZINC000096077628) in the αvβ3 binding site. The molecules are depicted using stick representation. The Mg^2+^ atom is shown as green sphere and docked structures are colored as follows: carbon (cyan), nitrogen (blue), oxygen (red), hydrogen (white), iodine (purple), and sulfur (yellow).

We next performed docking to a library of 26 thyroid hormones and their metabolites and two RGD ligands (cyclic and linear peptides). The free energies for the molecules in our model are summarized in **Table 1**. The best four scoring molecules (lowest Amber score) are depicted in **Figure 2A**, and the worst four scoring molecules (highest Amber score) are depicted in **Figure 2B**. The prohormone T4 and the biologically active hormone T3, are among top scoring compounds, substantiating previously published work which positioned both molecules as key αvβ3 integrin ligands (17). Notably, all the best four ranking molecules have a carboxyl group attached to the inner ring *via* two-carbon linker, while all the worst four ranking molecules are missing this group. The docked structure of sulfated T4 (T4S) with an amber score of -69.37 and decarboxylated T4 (T4AM) with an amber score of -21.19, are presented in **Figures 3A, B**, respectively. T4S interacts with one Mg_2+_ atom of the MIDAS sites through the carboxylic group and with another Mg^2+^ atom through the sulfate group, while T4AM does not closely interact with any of the Mg^2+^ atoms. This results in a reverse binding orientation of the two molecules. The docked structures of the best four scoring molecules are depicted in **Figure 3C** using wire representation, while the negatively charged oxygens of the carboxylic and sulfate groups are presented as red spheres. These negatively charges oxygens form electrostatic interactions with the Mg^2+^ atoms at the MIDAS site (green spheres). Thus, our model demonstrates the central role of metal ions as essential to thyroid hormones binding. Support that the negatively charged sulfates increase binding affinity to the integrin is provided by the higher amber score for sulfated T4 (T4S) and 3,3’T2 (3,3’T2S) in comparison to the non-sulfated hormones (**Table 1**). However, an exception was observed for T3S, in which amber score was higher compared to the non-sulfated metabolite, with the carboxylic group flipped away from the MIDAS site. Docking for T4 and T3 compared to their sulfated forms is depicted in **Figure 4**.

**Table 1:** Grid Amber score for thyroid hormone metabolites.

Molecule	Grid_Score kcal/mol	Amber_Score kcal/mol
ZINC000031706818 (3,3’ T2S, sulfated)^1^	-68.63	-76.90
ZINC000096077628 (T4S, sulfated)	-76.41	-69.37
RGDc^2^	-81.85	-58.30
ZINC000003830999 (3,3’, 5 T3, triiodothyronine)	-53.40	-51.72
ZINC000003830993 (T4, thyroxine)	-51.76	-42.96
ZINC000004097417 (reverse T3)	-53.19	-42.84
ZINC000085552312 (T3S, sulfated)	-65.81	-42.20
ZINC000013681007 (3T1AM, decarboxylated)	-40.60	-41.22
ZINC000085627494 (3’,5’ T2)	-59.25	-40.80
ZINC000016051523 (3,3’ T2)	-52.95	-40.33
ZINC000013681015 (3’,5’ T2AM, decarboxylated)	-42.16	-37.02
ZINC000006092925 (3’-T1)	-51.31	-35.13
ZINC000002387178 (3-T1)	-45.79	-35.01
ZINC000003922521 (RGD)^3^	-89.10	-33.82
ZINC000008681598 (tetrac, deaminated)	-63.63	-29.13
ZINC000004258247 (3,5 T2)	-46.02	-28.90
ZINC000000001575 (MIT - 3 iodotyrosine)	-45.15	-28.58
ZINC000003861723 (DIT - 3,5 Diiodothyrosine)	-54.26	-25.85
ZINC000028569157 (T3AM, decarboxylated)	-43.49	-25.64
reverse T3S (reverse T3, sulfated)	-67.65	-24.77
ZINC000000403598 (T0 - thyronine)	-46.88	-24.42
ZINC000004217580 (triac, deaminated)	-61.18	-24.15
ZINC000013681013 (3,3’-T2AM, decarboxylated)	-45.96	-23.61
ZINC000013681010 (3,5 T2AM, decarboxylated)	-41.32	-23.17
ZINC000013681005 (T0AM, decarboxylated)	-39.78	-22.47
ZINC000028567742 (reverse T3AM, decarboxylated)	-44.74	-22.23
ZINC000013681017 (3’-T1AM, decarboxylated)	-43.50	-22.21
ZINC000095606811 (T4AM – decarboxylated)	-44.02	-21.19

^1^ZINC database ID and common name in parentheses are given for each molecule whenever possible.

^2^cyclic pentapeptide Arg-Gly-Asp-{D-Phe}-{N-methyl-Val-}s.

^3^linear Arg-Gly-Asp peptide.

**Figure 5:**
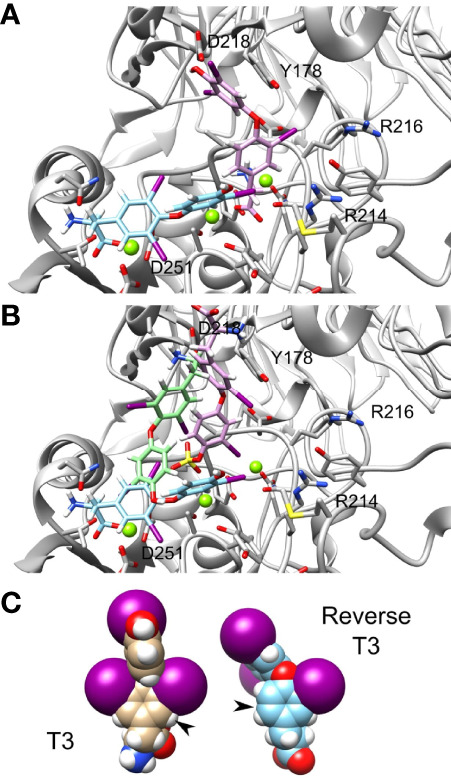
T3 and reverse T3 docked structures. Integrin’s binding site backbone (ribbons) and side chains (sticks) are colored gray with oxygen atoms in red and nitrogen in blue. **(A)** The docked structure of T3 (ZINC000003830999) and reverse T3 (ZINC000004097417) in the αvβ3 binding site. Atom coloring scheme is as follows: T3 carbon (cyan), reverse T3 carbon (pink), iodine (purple), oxygen (red), nitrogen (blue), and hydrogen (white). **(B)** Docked structures of T3, T3S (pink), and T3AM (green). Carbon colors are: T3 (cyan), T3S (pink), and T3AM (green). **(C)** Space filling model of T3 and reverse T3.

We further observed that thyroid hormones have increased affinity compared to their reverse form. Reverse T3 (rT3) is the metabolically inactive form of T3. In our calculation we show that the Amber score of T3 is -51.72 while that of reverse T3 is -42.84. Thus, reverse T3 has a smaller affinity to integrin αvβ3 binding site compared with that of T3. The docking poses of T3 (cyan) and reverse T3 (pink) are presented in **Figure 5A**, demonstrating different binding conformations. Despite the different poses, there is a steric clash between the T3 and reverse T3 docked structures. Thus, a competitive binding of the two hormones to the integrin αvβ3 binding site is postulated. Similarly, the Amber score of T3S is -42.20 and that of reverse T3S is -24.77 and the Amber score of T3AM is -25.64 and that of reverse T3AM -22.23. That is, in general iodination in positions 3,5, and 3’ increase the affinity of the hormone compared to iodination at positions 3, 3’, and 5’. **Figure 5B** shows the docked structures of T3 (cyan), T3S (pink), and T3AM (green). These three molecules have different binding poses to the integrin αvβ3 site, making the hypothesis that iodination in positions 3,5, and 3’ results in a better fit of these molecules to the binding site less likely. Another possible explanation is that iodination in positions 3,5, and 3’results in improved intrinsic binding ability. Space filling model of T3 and reverse T3 are presented in **Figure 5C**. The two iodine atoms in positions 3 and 5 of T3 on the inner ring tether the outer ring and restrict its rotation ability, along the single bonds connecting the two rings, as their size is comparable to the size of the ring itself. While the two iodine atoms in position 3’ and 5’ of the outer ring do not restrict its rotation ability along the single bonds connecting the two rings. Thus, iodine atoms at positions 3 and 5 rigidify the hormones. If this rigidification locks the molecules in their bound conformation it can increase their affinity to the receptor.

**Figure 6:**
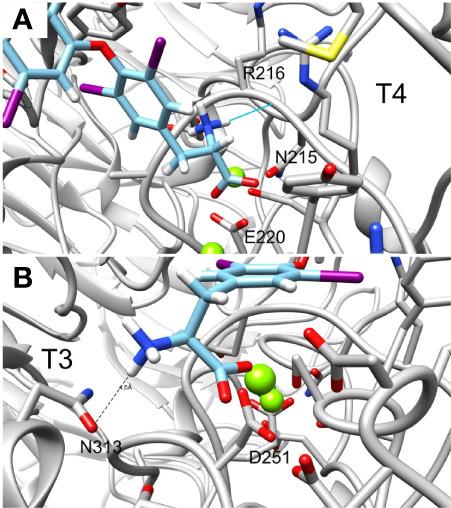
Close up of the amine and carboxylic groups of T3 and T4 docked structures. Integrin’s binding site backbone (ribbons) and side chains (sticks) are colored gray with oxygen atoms in red and nitrogen in blue. **(A)** T4 amine group forms hydrogen bond with N215 backbone carbonyl while the carboxyl group form electrostatic interaction with Mg^2+^ atom. **(B)** Similarly, T3 amine group form electrostatic interaction with N313 and the carboxyl group with Mg^2+^. Docked structures are colored as follows: carbon (cyan), nitrogen (blue) oxygen (red), hydrogen (white), and iodine (purple).

Lastly, the deaminated forms of T3 (triac) and T4 (tetrac) display higher amber scores compared to the native hormones (**Table 1**). In details, while T3 and T4 have amber scores of -51.72 and -42.96, respectively, the calculated score of triac and tetrac was -24.15 and -29.13, respectively. This suggests a role for the amine group in the interaction with the integrin receptor. **Figures 6A, B** show in close up the anime and carboxyl groups of T4 and T3, respectively. The carboxylic group of T4 contact Mg^2+^ atom while the amine group form hydrogen bond with the backbone carbonyl of N215. Similarly, the carboxyl group of T3 contact another Mg^2+^ atom while the amine group form electrostatic interaction with N313. Thus, the two groups contribute to the binding affinity of T4 and T3.

## Discussion

For many years thyroid hormones were considered to act *via* genomic actions, initiated by direct binding of the biologically active hormone T3 to its nuclear thyroid receptors (15). For these activities, T4 has significant lower affinities and is considered merely a prohormone. This dogma was challenged once a discrete binding site for both T3 and T4 was discovered upon a membrane receptor with multiple roles in physiology and malignancies, namely the αvβ3 integrin (17). This discovery was the first to demonstrate that small molecules can serve as ligands for this integrin. Since then, ample preclinical data in cell lines and animal models confirmed an array of functional consequences for this novel non-genomic signaling pathway (48). This highlights the importance of developing drugs targeting the hormone binding site upon the integrin.

Direct attachment of both T3 and T4 was validated not only by binding experiments with purified αvβ3 protein (20, 30, 49), but also by computational modeling. In this current study the binding conformations of the various hormone metabolites relative to the RGD binding pocket, revealed that T3 and its decarboxylated (T3AM) and sulfated (T3S) forms bind at close proximity to the RGD pocket. The results for T3 are supported by experimental work indicating that RGD hinders T3 binding to its exclusive S1 site upon the αvβ3 integrin (49). In contrast, T4 and rT3, which similarly affects cancer proliferation *via* the integrin (50), are located at a different binding pocket which is farther from the RGD site. Notably, decarboxylated T4 (T4AM) binding also overlaps with a site close to the RGD pocket, while its sulfated form (T4S) intersects with both the RGD and T4 sites. Combined with their low calculated free energy and expected lack of activity (8, 9), this suggests that the sulfated forms of T4 and T3 may act as novel potential blockers for the RGD and hormone integrin sites. Cody at al. mapped binding of T4 and its deaminated form, tetrac, to the RGD site in the integrin pocket (35). This preliminary modeling study also indicated that due to the smaller overall length of the thyroid hormone compared with the RGD peptide, the hormone can occupy the RGD binding site, mostly by interacting with the βA domain in the extracellular domain of the integrin, and not *via* the Arg recognition site in the αv propeller domain. A later study further combined quantum mechanical-molecular mechanical (QM/MM) molecular dynamics simulations and characterized the intermolecular interactions for T3, T4, tetrac and a series of hormone analogues (27). These data revealed that T3, T4 and tetrac may occupy two alternate sites in the integrin RGD binding pocket. In one orientation of the T4, the 4’-hydroxyphenyl ring occupies a binding pocket deeper within the crevice between the αvβ3 domains, while in the other binding mode, the phenolic ring occupies the interface between the integrin αvβ3 domains. For T3 the tyrosyl moiety may lay along the Arg side chain of the cyclic RGD peptide, or places the 4’-phenolic ring in a different binding pocket than observed for T4. The results for tetrac reveal one orientation which aligns similar to T3 along the side chain of Arg in the RGD peptide binding site, while in the other binding mode tetrac’s phenolic ring binds deeper within the RGD binding pocket, similar to that of T4.

The biological thyroid hormones, T3 and T4, were among the molecules with the lowest calculated interaction energy with αvβ3. This, together with the observation that the majority of T3 and T4 catabolized metabolites display significantly reduced binding affinity to the integrin, substantiates that the major integrin ligands are the native thyroid hormones. Based on the calculated free energy, T3 has higher affinity to the integrin compared to T4. These results are in accord with Freindorf et al., who suggested that an additional iodine atom in T4 may affect its interaction with the protein (27). However, as the prohormone T4 is produced at significantly higher physiological concentrations compared to T3 (13), it is positioned as the major endogenous ligand for the αvβ3 integrin. This was confirmed by binding assays using physiological and supra physiological concentrations of both hormones (20, 30, 49).

The best ranking molecules in our model are carboxylated, while the worst ranking molecules lack this group. This result may be explained by the interaction of the carboxylic group with the Mg^2+^ atom in the integrin MIDAS site, which is absent in the decarboxylated compounds. Moreover, the top binding compounds were sulfated forms of 3,3’ T2 (T2S) and T4 (T4S). *In silico* docking for these best scoring compounds demonstrated that the negatively charged oxygen of the sulfate group interacts with another Mg^2+^ atom of the MIDAS sites. This may also explain the reverse orientation between sulfated T4 (T4S) and decarboxylated T4 (T4AM), as well as that of sulfated T3 in comparison to the non-sulfated form. The addition of sulfate at position 4’ enable the molecule to bind *via* its negatively charge sulfate and carboxylic groups with the Mg^2+^ atoms. Removal of these two negative groups at T4AM results in a change of orientation that moves the positively charged amine group away from the Mg^2+^ atoms and bring it close to Asp218 and Asp219 to contacts these negatively charges amino acids. Collectively, our results provide support that the metal ions in the αvβ3 integrin domain are essential to thyroid hormone binding. This is in accordance with the well-defined characteristic of integrins, in which ligand binding is dependent upon bivalent cations (25, 26). However, the effect of sulfate on interaction with the integrin MIDAS domain should be further elucidated, as sulfated T3 did not display increased affinity compared to the non-sulfated hormone. We further observed that iodine localization in the hormones affects binding affinity to the integrin. Reverse forms of T3, sulfated T3 and decarboxylated T3 demonstrated significantly higher free energy compared to their native forms. Based on space filling model for T3 and reverse T3 we identified that the iodine atoms in positions 3 and 5 on the inner ring restrict rotation ability and rigidify and locks the hormone bound conformation. This results in increased affinity to the integrin receptor. In contrast, the two iodine atoms in position 3’ and 5’ of the outer ring, observed in the reverse forms, do not display such capabilities. Lastly, the deaminated forms of T3 (triac) and T4 (tetrac) present lower affinity to the integrin compared with the native hormones. This observation, which was reported before by Freindorf et al. (27), stresses the importance of the NH2 group in the interaction between the ligands and the integrin.

To conclude, by performing a comprehensive three-dimensional docking for thyroid hormone and an array of naturally occurring endogenous metabolites, we have identified that their binding to the αvβ3 integrin is affected by several structure-related characteristics. As the involvement of the thyroid hormone-integrin axis in cancer progression and inhibition is gaining more attention, this study may lead to better understanding of the tumor promoting effects of various thyroid dysfunction conditions, as well as to the development of effective inhibitors to the integrin site.

## Author’s Note

This article is dedicated to the memory of Dr Eilon Krashin, a brilliant physician, researcher and a friend.

## Data Availability Statement

The original contributions presented in the study are included in the article/**Supplementary Material**. Further inquiries can be directed to the corresponding authors.

## Author Contributions

DT, EK, and OA-F designed this study. DT performed the docking. DT, ME, PD, VC, and OA-F wrote, read and approved the manuscript.

## Conflict of Interest

The authors declare that the research was conducted in the absence of any commercial or financial relationships that could be construed as a potential conflict of interest.

## Publisher’s Note

All claims expressed in this article are solely those of the authors and do not necessarily represent those of their affiliated organizations, or those of the publisher, the editors and the reviewers. Any product that may be evaluated in this article, or claim that may be made by its manufacturer, is not guaranteed or endorsed by the publisher.
