# Supplementary material for: Three-Dimensional Modeling of Thyroid Hormone Metabolites Binding to the Cancer-Relevant αvβ3 Integrin: In-Silico Based Study
Source: Front Endocrinol (Lausanne). 2022 May 27;13:895240. doi: 10.3389/fendo.2022.895240 (PMC9186291; doi:10.3389/fendo.2022.895240)
Supplement: Supplementary file 1 [file DataSheet_1.pdf]

Figure S1: Dataset of 26 thyroid hormones and their metabolites

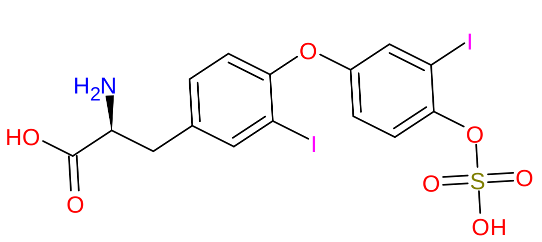

ZINC000031706818 (3,3' T2S)

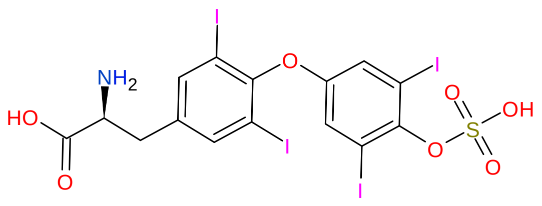

ZINC000096077628 (T4S)

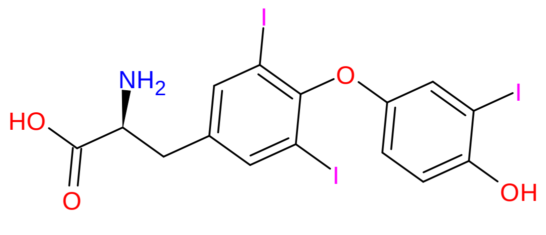

ZINC000003830999 (T3)

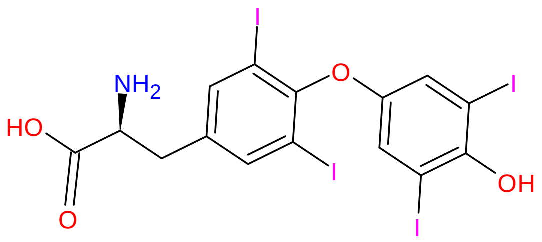

ZINC000003830993 (thyroxine)

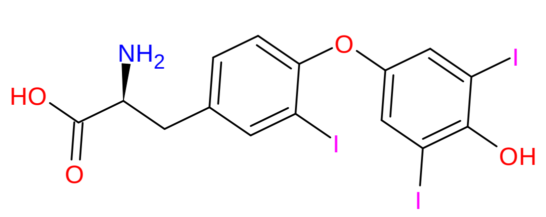

ZINC000004097417 (reverse T3)

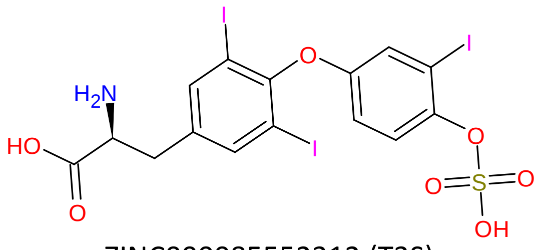

ZINC000085552312 (T3S)

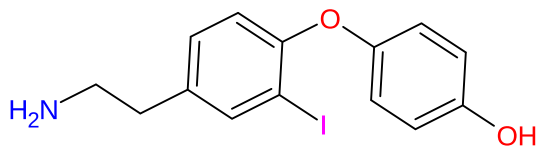

ZINC000013681007 (3-T1AM)

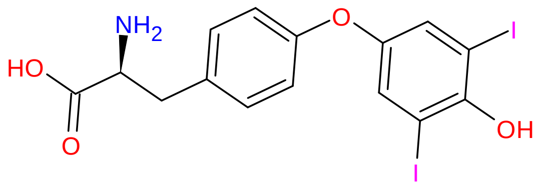

ZINC000085627494 (3',5' T2)

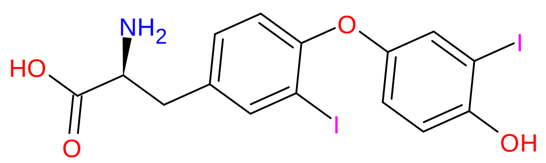

ZINC000016051523 (T2 - 3,3' Diiodothyronine)

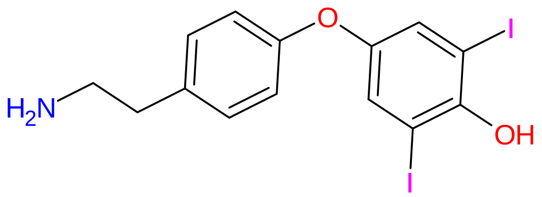

ZINC000013681015 (3',5' T2AM)

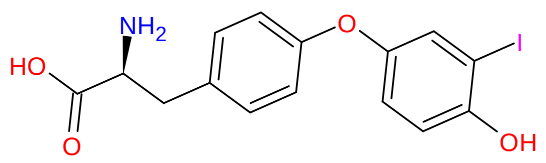

ZINC000006092925 (3'-T1)

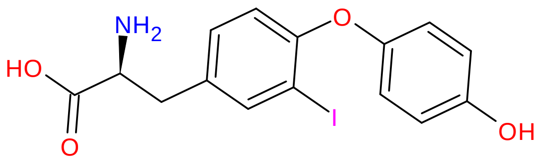

ZINC000002387178 (3-T1)

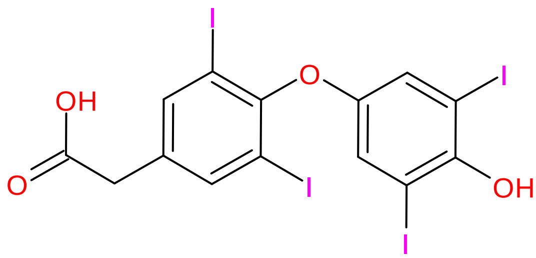

ZINC000008681598 (tetrac)

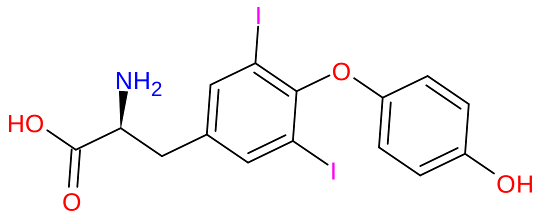

ZINC000004258247 (3,5 T2)

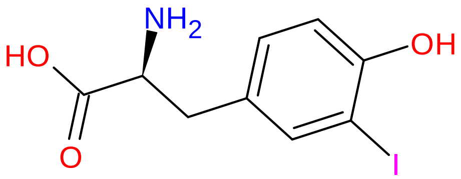

ZINC000000001575 (MIT - 3 iodotyrosine)

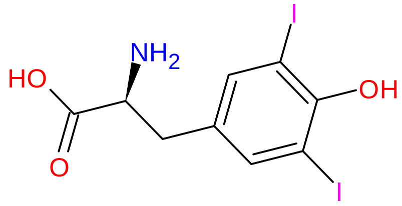

ZINC000003861723 (DIT - 3,5 Diiodothyrosine)

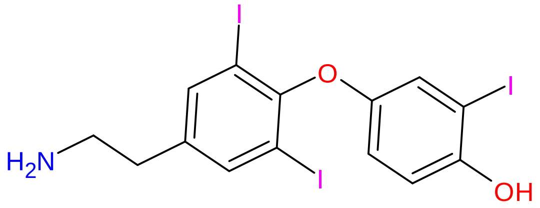

ZINC000028569157 (T3AM)

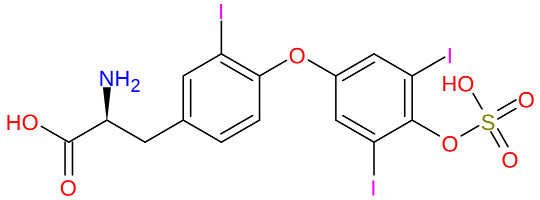

reverse T3S

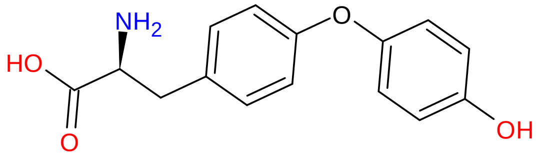

ZINC000000403598 (T0 - thyronine)

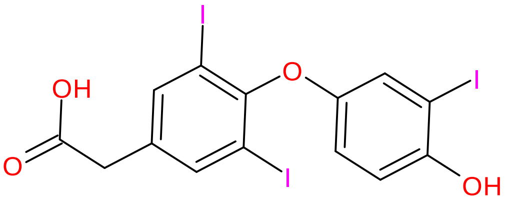

ZINC000004217580 (triac- tiratricol)

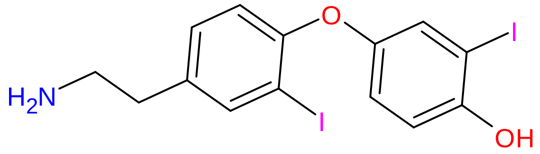

ZINC000013681013 (3,3'-T2AM)

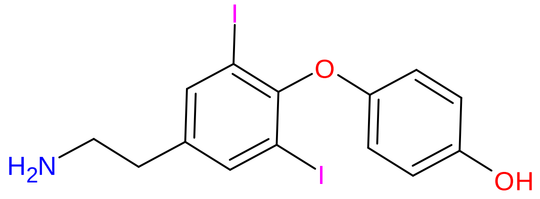

ZINC000013681010 (3,5 T2AM)

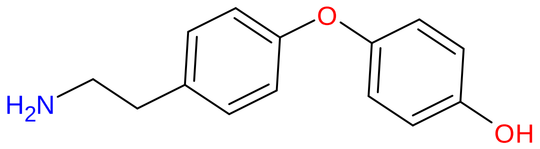

ZINC000013681005 (T0AM - thyronamine)

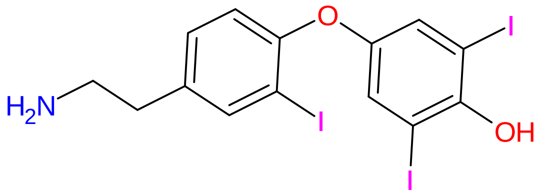

ZINC000028567742 (reverse T3AM)

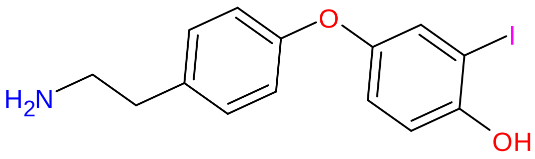

ZINC000013681017 (3'-T1AM)

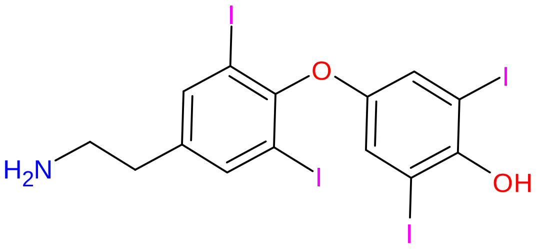

ZINC000095606811 (T4AM - decarboxylated thyroxine)
